# COVID-19 Vaccination Coverage in Italy: How Many Hospitalisations and Related Costs Could Have Been Saved If We Were All Vaccinated?

**DOI:** 10.3389/fpubh.2022.825416

**Published:** 2022-03-03

**Authors:** Giulia Zamagni, Benedetta Armocida, Cristiana Abbafati, Luca Ronfani, Lorenzo Monasta

**Affiliations:** ^1^Clinical Epidemiology and Public Health Research Unit, Institute for Maternal and Child Health—IRCCS “Burlo Garofolo”, Trieste, Italy; ^2^Department of Juridical and Economic Studies, Sapienza University of Rome, Rome, Italy

**Keywords:** COVID-19, COVID-19 vaccination, public health, Italy, COVID-19 response

## Background

The first cases of severe acute respiratory syndrome coronavirus 2 (SARS-CoV-2) infection were reported in December 2019. On March 11, 2020, the WHO declared coronavirus disease 2019 (COVID-19) a pandemic (1). By the end of 2021, COVID-19 has caused 5.4 million deaths worldwide, impacting severely on health systems and triggering a global economic and social crisis.

On November 24, 2021, Europe is once again the epicenter of the COVID-19 pandemic (2), with 75% of fatal cases occurring in people aged 65 years and above, and hospital admission rates more than doubling in 1 week, according to the latest data (2). The reasons behind this increase are mainly insufficient vaccination coverage and relaxation of public health and social measures (2).

Vaccination has been shown to be 70–95% effective against COVID-19 after two doses and against COVID-19-related hospital admissions up to 6 months after being fully vaccinated (3–5) and is considered one of the most cost-effective interventions to preserve healthcare resources and system efficiency (6). Yet, vaccination coverage within Europe is still suboptimal (2).

As of November 24, 2021, Italy has administered 95,571,957 doses of COVID-19 vaccines, with an 84.5% coverage of fully vaccinated population over 12 years old, and 87.3% with at least one dose (7). Four different vaccines (Cominarty, Spikevax, Vaxzevria, and Janssen) have been administered since December 27, 2020. Up until September 26, 2021, 71.2% of administered doses were Cominarty, 14.5% were Vaxzevria, 12.5% were Spikevax, and 1.8% were Janssen (8). Since September 27, 2021, the booster dose, administered right after the date of vaccination only of Cominarty and Spikevax, as well as the second dose of Janssen, was initially indicated only for people aged over 80 years, as well as residents and staff of nursing homes and healthcare facilities. It was subsequently extended to all subjects vaccinated with a single dose of Janssen vaccine from at least 6 months, regardless of age, and has now been extended to the whole over-18 population, provided a minimum of 5 months have passed since the completion of the primary course with two doses (9–11). Despite these efforts and the establishment of a COVID-19 Green Pass, which allows access to public events, transportations, and nursing homes only to vaccinated people or people tested for SARS-CoV-2 (12), trends of hospital admissions due to COVID-19 are rising, particularly among the non-vaccinated who amount to 8 million people. Indeed, as of November 24, 2021, data provided by the Italian National Health Institute (ISS) show an increased risk of hospital admission and death for the non-vaccinated population in all age groups, with new cases primarily caused by the B.1.617.2 (Delta) variant of SARS-CoV-2 (13). In the light of this situation, the Italian Ministry of Health (MoH) instituted a reinforced COVID-19 Green Pass that allowed only people who are fully vaccinated or recovered from COVID-19 to access shows, sporting events, indoor bars and restaurants, parties and discos, public ceremonies for the period December 6, 2021 to January 15, 2022 in the Italian white zones, and from November 29, 2021 to the end of the state of emergency in the yellow and orange zones (14). Moreover, mandatory vaccination has been extended to new categories (e.g., school and police personnel) and the validity of the COVID-19 Green Pass has been reduced from 12 to 9 months (14).

Using the official data from ISS for the period October 24-November 24, 2021, on vaccination status, new positives, hospitalisations in general wards and intensive care units, this brief report aims to: (i) to assess the risks of hospital admission for different age groups ≥12 years of age, and by vaccination status; (ii) to calculate the costs of vaccine refusal during the observation period.

The costs of excess cases due to vaccine refusal were calculated only for hospitalization. Neither the direct and indirect costs of non-hospitalized cases nor the cost of deaths, which however we believe to be significant, were factored into the calculation.

## Methods

Data were retrieved from the ISS COVID-19 Report and referred to a time period of 30 days, updated to November 24, 2021 (13). The study population, which included subjects 12 years of age and above, was divided into four age groups (12–39, 40–59, 60–79 years, and over 80 years), and by vaccination status (unvaccinated, vaccinated with one dose, fully vaccinated <6 months from the second dose, fully vaccinated more than 6 months from the second dose, vaccinated with the booster dose). The number of new cases, hospitalisations in ordinary wards and intensive care units (ICUs) are presented by age group and vaccination status.

Based on the estimates provided by the Regional Agency of Health (ARS) Toscana, we determined the average length of stay to be 11 days in ordinary wards, and 12 days in ICUs (15) the mean daily cost of hospitalization per person being €710 in a general ward and €1,680 in the ICU (16).

The relative risks (RRs) of infection, hospitalization, and ICU admission were computed for the unvaccinated vs. each of the other vaccination status categories. Studies show the vaccine's efficacy in preventing infections and hospitalisations wanes after 6 months (17, 18), hence the RRs were reported separately for subjects who had completed the vaccination cycle more than 6 months earlier, for those who had completed it within the last 6 months and for those who had received the booster dose. Stratified RRs were also computed considering the four ages groups.

In addition, we calculated the number of hospital admissions, as well as related costs, that could have been saved if the entire population were vaccinated. More specifically, the number of excess cases and hospitalizations due to vaccination refusal was calculated using as a reference the event rates among fully vaccinated individuals who had completed the vaccine cycle within the last 6 months. We considered this to be the most stable group at a time in which the booster dose was being administered only to a small proportion of the population, consisting mainly of vulnerable subjects and professional categories at high risk of exposure, such as healthcare personnel.

## Results

On November 24, over 7.5 million Italians aged 12 years and above had not been vaccinated, and more than 2 million people had not completed the vaccination course. Overall, fully vaccinated persons were more than 44 million, among which 38.6 million received their second dose within 6 months, 4.5 million with the second dose beyond 6 months, and 1 million had received the booster dose.

Data and analysis results are reported in Table 1. The risk of being infected by SARS-CoV-2 was 3.9 times higher for the unvaccinated compared to individuals who had received the second dose of vaccine within the last 6 months. A non-vaccinated person had a RR of 7.3 of being hospitalized, of 22.7 of being admitted to an ICU, and a six times higher risk of dying.

**Table 1:** Hospitalisations and deaths due to coronavirus disease 2019 (COVID-19), by age groups and vaccination status, nearing 30 days to November 24, 2021.

**Age groups and vaccination status**	**Overall population**	**New positives (per 100,000)**	**New hospitalisations in ordinary wards (per 1,000 confirmed cases)**	New hospitalisations in ICUs^*^(per 1,000 confirmed cases)	**Deaths (per 1,000 confirmed cases)**
**12–39 years of age**					
Unvaccinated	3,271,713	28,535 (872.17)	505 (17.70)	23 (0.81)	7 (0.25)
Complete cycle, past 6 months	638,196	2,910 (455.97)	33 (11.34)	0 (0.00)	0 (0.00)
Risk ratio^**^		1.91 (1.84–1.99)	2.99 (2.10–4.25)	–	–
Incomplete cycle	1,087,094	1,730 (159.14)	25 (14.45)	0 (0.00)	0 (0.00)
Risk ratio^**^		5.48 (5.22–5.75)	6.70 (4.49–10.03)	–	–
Complete cycle, within 6 months	12,382,786	23,907 (193.07)	121 (5.06)	0 (0.00)	1 (0.04)
Risk ratio^**^		4.52 (4.44–4.60)	15.80 (12.95–19.26)	–	–
Complete cycle, including booster dose	55,766	90 (161.39)	1 (11.11)	0 (0.00)	0 (0.00)
Risk ratio^**^		5.40 (4.39–6.65)	8.61 (1.21–61.23)	–	–
Avoidable cases^***^		22,218	473	23	7
**40–59 years of age**					
Unvaccinated	2,832,244	23,381 (825.53)	1,251 (53.50)	153 (6.54)	49 (2.10)
Complete cycle, past 6 months	1,030,331	4,584 (444.91)	81 (17.67)	3 (0.65)	3 (0.65)
Risk ratio^**^		1.86 (1.80–1.92)	5.62 (4.49–7.03)	18.55 (5.92–58.16)	5.94 (1.85–19.06)
Incomplete cycle	774,605	1,660 (214.30)	35 (21.08)	3 (1.81)	1 (0.60)
Risk ratio^**^		3.85 (3.67–4.05)	9.78 (6.99–13.68)	13.95 (4.45–43.73)	13.4 (1.85–97.05)
Complete cycle, within 6 months	13,640,735	32,369 (237.30)	325 (10.04)	19 (0.59)	14 (0.43)
Risk ratio^**^		3.48 (3.42–3.54)	18.54 (16.41–20.94)	38.78 (24.08–62.47)	16.86 (9.31–30.53)
Complete cycle, including booster dose	155,336	220 (141.63)	5 (22.73)	1 (4.55)	0 (0.00)
Risk ratio^**^		5.83 (5.10–6.66)	13.72 (5.7–33.03)	8.39 (1.17–59.95)	–
Avoidable cases^***^		16,660	1,184	149	46
**60–79 years of age**					
Unvaccinated	1,254,000	8,231 (656.38)	1,369 (166.32)	298 (36.20)	193 (23.45)
Complete cycle, past 6 months	779,291	2,932 (376.24)	174 (59.35)	20 (6.82)	16 (5.46)
Risk ratio^**^		1.74 (1.67–1.82)	4.89 (4.18–5.72)	9.26 (5.89–14.56)	7.50 (4.50–12.48)
Incomplete cycle	272,933	701 (256.84)	70 (99.86)	10 (14.27)	5 (7.13)
Risk ratio^**^		2.56 (2.37–2.76)	4.26 (3.35–5.41)	6.49 (3.45–12.18)	8.40 (3.46–20.41)
Complete cycle, within 6 months	11,051,025	22,710 (205.50)	1,281 (56.41)	151 (6.65)	108 (4.76)
Risk ratio^**^		3.19 (3.11–3.28)	9.42 (8.73–10.16)	17.39 (14.3–21.15)	15.75 (12.44–19.93)
Complete cycle, including booster dose	215,530	258 (119.70)	31 (120.16)	1 (3.88)	0 (0.00)
Risk ratio^**^		5.48 (4.84–6.21)	7.59 (5.32–10.84)	51.22 (7.17–364.81)	–
Avoidable cases^***^		5,654	1,224	281	181
**>** **80 years of age**					
Unvaccinated	228,056	1,761 (772.18)	612 (347.53)	35 (19.88)	200 (113.57)
Complete cycle, past 6 moths	2,078,888	5,093 (244.99)	899 (176.52)	35 (6.87)	172 (33.77)
Risk ratio^**^		3.15 (2.99–3.33)	6.21 (5.60–6.88)	9.12 (5.71–14.56)	10.60 (8.65–13.00)
Incomplete cycle	88,040	169 (191.96)	52 (307.69)	1 (5.92)	10 (59.17)
Risk ratio^**^		4.02 (3.44–4.71)	4.54 (3.42–6.03)	13.51 (1.85–98.63)	7.72 (4.09–14.57)
Complete cycle, within 6 months	1,584,000	2,754 (173.86)	710 (257.81)	39 (14.16)	236 (85.69)
Risk ratio^**^		4.44 (4.18–4.71)	5.99 (5.37–6.67)	6.23 (3.95–9.84)	5.89 (4.88–7.11)
Complete cycle, including booster dose	589,375	401 (68.04)	32 (79.80)	1 (2.49)	5 (12.47)
Risk ratio^**^		11.35 (10.18–12.65)	49.43 (34.64–70.52)	90.45 (12.39–660.25)	103.37 (42.56–251.08)
Avoidable cases^***^		1,364	510	29	166
**Total population**					
Unvaccinated	7,586,013	61,908 (816.08)	3,737 (60.36)	509 (8.22)	449 (7.25)
Complete cycle, past 6 moths	4,526,706	15,519 (342.83)	1,187 (76.49)	58 (3.74)	191 (12.31)
Risk ratio^**^		2.38 (2.34–2.42)	1.88 (1.76–2.01)	5.24 (3.99–6.87)	1.40 (1.18–1.66)
Incomplete cycle	2,222,672	4,260 (191.66)	182 (42.72)	14 (3.29)	16 (3.76)
Risk ratio^**^		4.26 (4.13–4.39)	6.02 (5.18–6.98)	10.65 (6.26–18.12)	8.22 (4.99–13.54)
Complete cycle, within 6 months	38,658,546	81,740 (211.44)	2,437 (29.81)	209 (2.56)	359 (4.39)
Risk ratio^**^		3.86 (3.82–3.90)	7.81 (7.43–8.22)	12.41 (10.57–14.58)	6.37 (5.55–7.32)
Complete cycle, including booster dose	1,016,007	969 (95.37)	69 (71.21)	3 (3.10)	5 (5.16)
Risk ratio^**^		8.56 (8.03–9.12)	7.25 (5.72–9.20)	22.72 (7.30–70.69)	12.03 (4.98–29.04)
Avoidable cases^***^		164,396	7,612	793	1,020

* *ICUs, intensive care units*.

** *Risk ratios are relative to the excess risk of an unvaccinated, compared to each other category*.

*** *Avoidable cases are calculated as if the proportion of cases for each category was the same as the one in the “Complete cycle, within 6 months”*.

In the comparison between the unvaccinated and subjects vaccinated with two doses, but after 6 months from the second dose, all RRs reduced by more than a half. However, in almost all age groups, the RRs of SARS-CoV-2 positivity and hospital and ICU admission still differed significantly. The administration of the booster dose sensibly reduced the risk of infection and severe COVID-19: a not vaccinated person aged >80 had a RR of being hospitalized and admitted to ICU of 49.4 and 90.45, respectively.

In the 30 days to November 24, 27.9% of new cases, 44.5% of ordinary hospitalizations, and 60.8% of ICU admissions could have been avoided if all the over-12 population had completed the primary vaccination course with two doses within the prior 6 months, which would have saved (the National Healthcare System) €36,198,929 of the €75,436,000 estimated total expense (48%). For every million fully vaccinated individuals, we would have saved 8,161 new cases, 493 ordinary hospitalisations, 67 ICU admissions, 59 deaths, and an overall expense for hospitalisations of € 5,200,019 in the last 30 days.

## Discussion

Our findings show that, during the period under investigation, the risk of being infected with SARS-CoV-2, hospitalized, admission in ICU, or death was much higher for the non-vaccinated compared to the fully vaccinated, thereby highlighting the importance of prioritizing COVID-19 vaccination. The impact of vaccines was even more pronounced when the vaccination cycle had been completed within the prior 6 months. This is in line with the literature, which reports a substantial reduction in humoral response after 6 months from the second dose, especially among men, among individuals 65 years of age or older, and among immunocompromised subjects (17, 18).

Nearing 30 days to November 24, we could have avoided 27.9% of cases, 44.5% of hospitalisations in ordinary wards, 60.8% hospitalisations in ICUs, and 39.2% of deaths. This excess was generated by the unvaccinated, which represented 14% of the population ≥12 years of age.

Although all the RRs were reduced by more than a half after 6 months from the completion of the full vaccination cycle, the risks for the non-vaccinated remained significantly higher.

Our results also reinforce the importance of the booster dose, currently recommended by the Italian MoH to all people ≥18 years of age after no <5 months from the second dose (9–11).

The effectiveness of the booster dose seems to be less marked in younger age groups when compared to the effectiveness of the second dose administered within the prior 6 months. This can probably be explained by the fact that the booster campaign among young people was only started at a later stage, and that the vulnerable subjects who made up most of the population who got booster shots at the time of data collection are more likely to develop severe illness, regardless of their vaccination status.

Studies have shown that besides being effective in terms of clinical outcome, COVID-19 vaccines are also able to reduce health care costs by 80% or more (19). This is in line with our findings, according to which the expenditure for ordinary hospitalizations and ICU admissions of unvaccinated individuals account for 48% of the total expenditure for all hospitalizations.

In a subsequent analysis conducted on data for the period 13th December 2021–12th January 2022, during which the curve of cases, hospitalisations, and deaths grew significantly, the percentage of avoidable cases, the RRs, and the proportion of avoidable costs of hospital admissions did not vary substantially. Indeed, the entity of the problem had changed: had all over-12 had been vaccinated during those 30 days period, 1,200 deaths, 181 thousand positives, 8,000 hospitalisations, and 1,300 ICU admissions would have been avoided, in which €90 million could have been saved. Unfortunately, a full comparison between the two observation periods is not possible due to intervening changes in ISS reporting criteria, whereby the safe COVID-19 vaccine duration of immunity was no longer considered to be 6 months, but 120 days.

Comorbidities, especially those related to chronic conditions, increase with age. Similarly, RRs had been drawn from the comparison between the unvaccinated vs. the vaccinated population, which also tend to reduce with age. Chronic illnesses are strongly related to adverse or more severe COVID-19 prognoses (20, 21), and this is true also for vaccinated subjects, although to a lesser extent compared to the unvaccinated.

Our study presents some limitations, due to the fact that the estimation of vaccination cost-effectiveness should take into account a wider range of costs than the one we considered (22), including the direct and indirect costs of disability, illness, long-COVID, quarantine, isolation, and workdays lost. Moreover, data on post-infection health status were not available for either the unvaccinated or the vaccinated population. Finally, due to the nature of available data, it was not possible to stratify the population by type of vaccine received in order to obtain additional information on the efficacy of each vaccine.

Compared to other European countries, Italy ranks among the top 10 countries with the highest vaccination coverage, which may explain why it is currently experiencing a milder burden of COVID-19 fourth wave. However, in the light of our findings, this is not sufficient. Should the rates of vaccination uptake in Europe remain at the current suboptimal level, the burden of COVID-19 over the winter will be enormous, especially in countries with lower vaccination coverage (23). Studies have also shown that it is essential to modulate the lifting of public health measurements of containment in accordance with the progress of the vaccination programs, to avoid increases in COVID-19 incidence and the risk of ICU admissions as a result of the premature lifting of restrictions (24).

Considering the impact of vaccine refusal both in terms of healthcare system overload and costs, it is pivotal that public health policies are directed to mitigating the burden of the fourth COVID-19 wave that is currently hitting Europe, by increasing vaccination coverage, concentrating efforts toward the widespread rollout of booster doses, and reintroducing preventive measures.

## Author Contributions

LM conceptualized the study. GZ and BA drafted the manuscript. LM and GZ provided the analysis. CA and LR contributed to reviewing and finalizing the manuscript. All authors approved the final version of the manuscript. All authors have contributed to preparing the manuscript and their authorship meets the International Committee of Medical Journal Editors (ICMJE) criteria.

## Conflict of Interest

The authors declare that the research was conducted in the absence of any commercial or financial relationships that could be construed as a potential conflict of interest.

## Publisher's Note

All claims expressed in this article are solely those of the authors and do not necessarily represent those of their affiliated organizations, or those of the publisher, the editors and the reviewers. Any product that may be evaluated in this article, or claim that may be made by its manufacturer, is not guaranteed or endorsed by the publisher.
